# Supplementary material for: Metagenomic Analysis of Plasma Microbial Extracellular Vesicles in Patients Receiving Mechanical Ventilation: A Pilot Study
Source: J Pers Med. 2022 Apr 2;12(4):564. doi: 10.3390/jpm12040564 (PMC9031263; doi:10.3390/jpm12040564)
Supplement: Supplementary file 1 [file jpm-12-00564-s001.zip › Supplementary Figure S1.pdf]

Supplementary Figure S1. ROC curves showing the accuracy of MEV strain to discriminate between survivors and non-survivors at day 7 AUC 0.70 (95% CI 0.63-0.80, *P*=0.126)

X1	Acinetobacter
X2	Methylobacterium

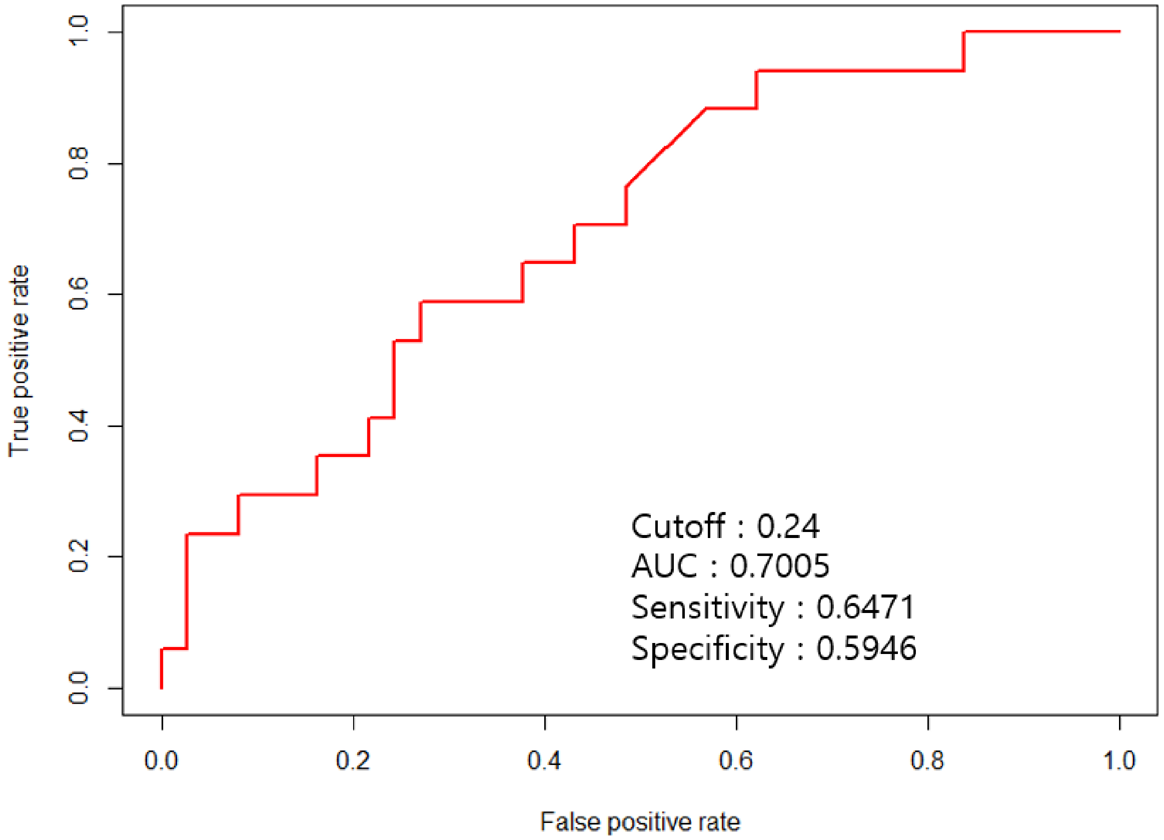

MEV: microbial extracellular vesicle; ROC: receiver operating characteristic; AUC: area under the receiver operating characteristic curve. CI confidence interval
