# Supplementary material for: Metagenomic Analysis of Plasma Microbial Extracellular Vesicles in Patients Receiving Mechanical Ventilation: A Pilot Study
Source: J Pers Med. 2022 Apr 2;12(4):564. doi: 10.3390/jpm12040564 (PMC9031263; doi:10.3390/jpm12040564)
Supplement: Supplementary file 1 [file jpm-12-00564-s001.zip › Supplementary Table S1.pdf]

Supplementary Table S1. Antibiotic therapy per group between day 1 and day 7

Antibiotic used between day 1 and day 7	Pneumonia n=41	Non-pneumonia n=18	*P*	NHAI n=24	Non NHAI n=35	*P*	Survivor n=41	Non-survivor n=18	*P*
b-lactams (only)	5 (12.2%)	8 (44.4%)	0.012	1 (4.2%)	12 (34.3%)	0.01	9 (22%)	4 (22.2%)	0.431
b-lactams plus fluoroquinolones	6 (14.6%)	3 (16.7%)		2 (8.3%)	7 (20%)		8 (19.5%)	1 (5.6%)	
b-lactam plus vancomycin	19 (46.3%)	7 (38.9%)		14 (58.3%)	12 (34.3%)		18 (43.9%)	8 (44.4%)	
b-lactam plus vancomycin plus other antibiotics (colistin or fluoroquinolones)	11 (26.8%)	0 (0%)		7 (29.2%)	4 (11.4%)		6 (14.6%)	5 (27.8%)	

NHAI: nursing home and hospital-associated infection;
